# Highly Selective Supported Graphene Oxide Membranes for Water-Ethanol Separation

**DOI:** 10.1038/s41598-019-38485-y

**Published:** 2019-02-19

**Authors:** Yongsoon Shin, Mohammad Fuad Nur Taufique, Ram Devanathan, Erika C. Cutsforth, Jaewon Lee, Wei Liu, Leonard S. Fifield, David W. Gotthold

**Affiliations:** Pacific Northwest National Laboratory, 902 Battelle Blvd, P.O.Box 999, Richland, Washington 99352 United States

## Abstract

A polyethersulfone (PES)-supported graphene oxide (GO) membrane has been developed by a simple casting approach. This stable membrane is applied for ethanol/water separation at different temperatures. The 5.0 µm thick GO film coated on PES support membrane showed a long-term stability over a testing period of one month and excellent water/ethanol selectivity at elevated temperatures. The water/ethanol selectivity is dependent on ethanol weight percentage in water/ethanol feed mixtures and on operating temperature. The water/ethanol selectivity was enhanced with an increase of ethanol weight percentage in water/ethanol mixtures, from below 100 at RT to close to 874 at a 90 °C for 90% ethanol/10% water mixture. Molecular dynamics simulation of water-ethanol mixtures in graphene bilayers, that are considered to play a key role in transport, revealed that molecular transport is negligible for layer spacing below 1 nm. The differences in the diffusion of ethanol and water in the bilayer are not consistent with the large selectivity value experimentally observed. The entry of water and ethanol into the interlayer space may be the crucial step controlling the selectivity.

## Introduction

Graphene oxide (GO) has attracted considerable interest in the areas of energy storage and separation technologies^1^. GO can form a stable aqueous suspension due to the presence of functionalities, such as carboxyl, hydroxyl, and epoxide groups^2^. In particular, GO sheets have shown great potential as materials for functional composite membranes that allow unimpeded permeation of water vapor^3,4^, while being totally impermeable to other gas molecules including helium^5^. Despite increased interest in water transport through graphene and GO membranes, significant technical difficulties still hinder the fabrication of such membranes for real-world water separation applications. Molecular simulations have predicted that water has a very large slip length (i.e., low friction) on graphene surfaces, resulting in an extremely high rate of water flow in planar graphene nanochannels^6,7^. This property promises high water flux in stacked GO nanosheets, but membrane instability during operation has been a challenge^8^. This work reports a scalable and stable casting of GO dispersion onto polyethersulfone (PES) membranes for water-ethanol separation, molecular-level insights into selective transport, and the feasibility of GO/PES membranes for dehydration of biofuels.

Bio-ethanol is the most commonly used biofuel (up to 10% in gasoline) in the United States. In 2015, the U.S. ethanol consumption was 14.061 billion gallons, and in 2017 it will be 14.370 billion gallons, where consumption has grown by about 400% compared with 2004^9^. To produce high purity (99%) ethanol from dilute fermentation broths (10–14 wt.% ethanol), fractionation, distillation and adsorption technologies are commonly used. These processes have the drawback that they require additional energy and expense to overcome the ethanol–water azeotrope formation^10^. Ethanol-selective and water-selective membrane technologies have been considered to replace the conventional distillation process for liquid–liquid separation^11^. Energy efficient and inexpensive separation of ethanol from water is a crucial step for potential bio-fuel applications^12^.

To date, GO-based membranes have been studied mainly for water and gas separation. For instance, Nair *et al*.^5^ evaluated the permeation rates of single-component vapors or gases. There are only a few papers focusing on the performance of membranes in separating water from water-alcohol mixtures^13,14^. For the separation of binary mixtures, the interaction between feed components and GO membranes is an inevitable factor that could affect separation efficiency. One of the main drawbacks for free-standing GO membranes is instability during the separation test due to the extremely hydrophilic character of GO^6–8^.

**Figure 1:**
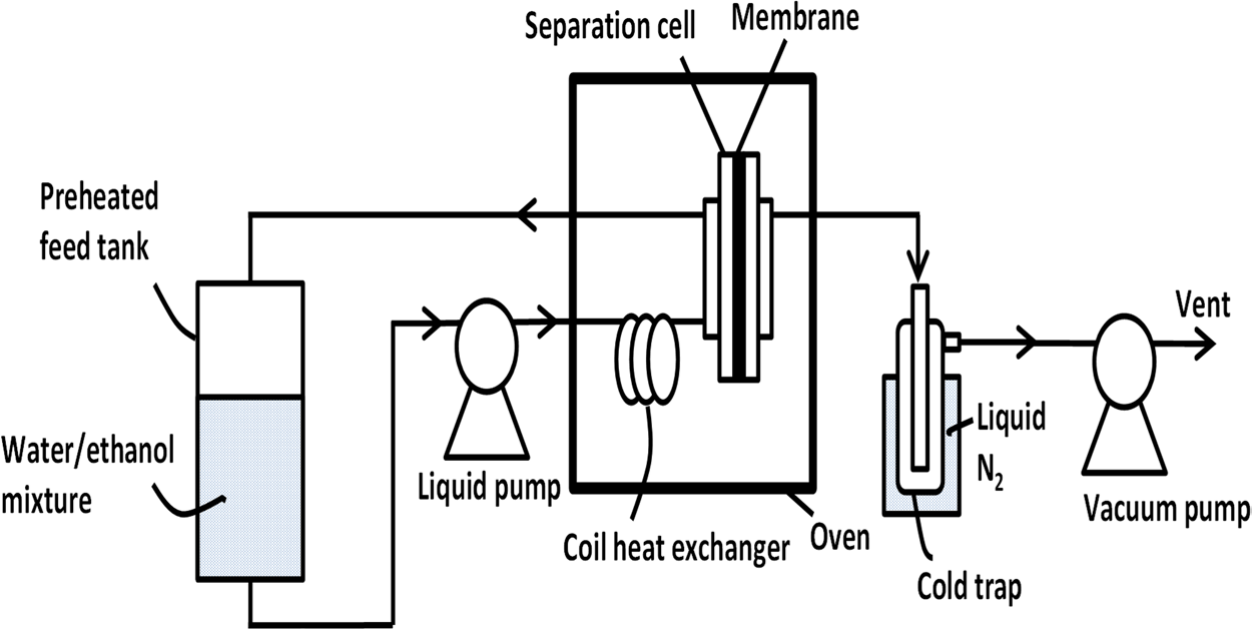
Schematic diagram of the water-ethanol separation test system.

The GO/PES membrane synthesized in this work was very stable up to a temperature of 120 °C during the water-ethanol (H_2_O-EtOH) separation test performed as shown in Fig. 1. The membrane showed impressive separation performance with high separation factors of H_2_O-EtOH at 90 °C. The H_2_O-EtOH selectivity for the GO/PES membrane increased, while the total flux dramatically decreased with increase in the wt.% of EtOH in the H_2_O-EtOH feed mixtures. Molecular dynamics (MD) simulations have shed light on the transport of H_2_O and EtOH in narrow graphene capillaries that are considered to play a key role in the GO membrane permeation properties^15,16^.

## Results and Discussion

Our focus is to examine preferential transport of water relative to ethanol through a GO/PES membrane that was prepared by a simple casting approach. The emphasis is on characterizing the influence of the feed composition and the separation temperature on selective molecular transport.

### Membrane Characterization

**Figure 2:**
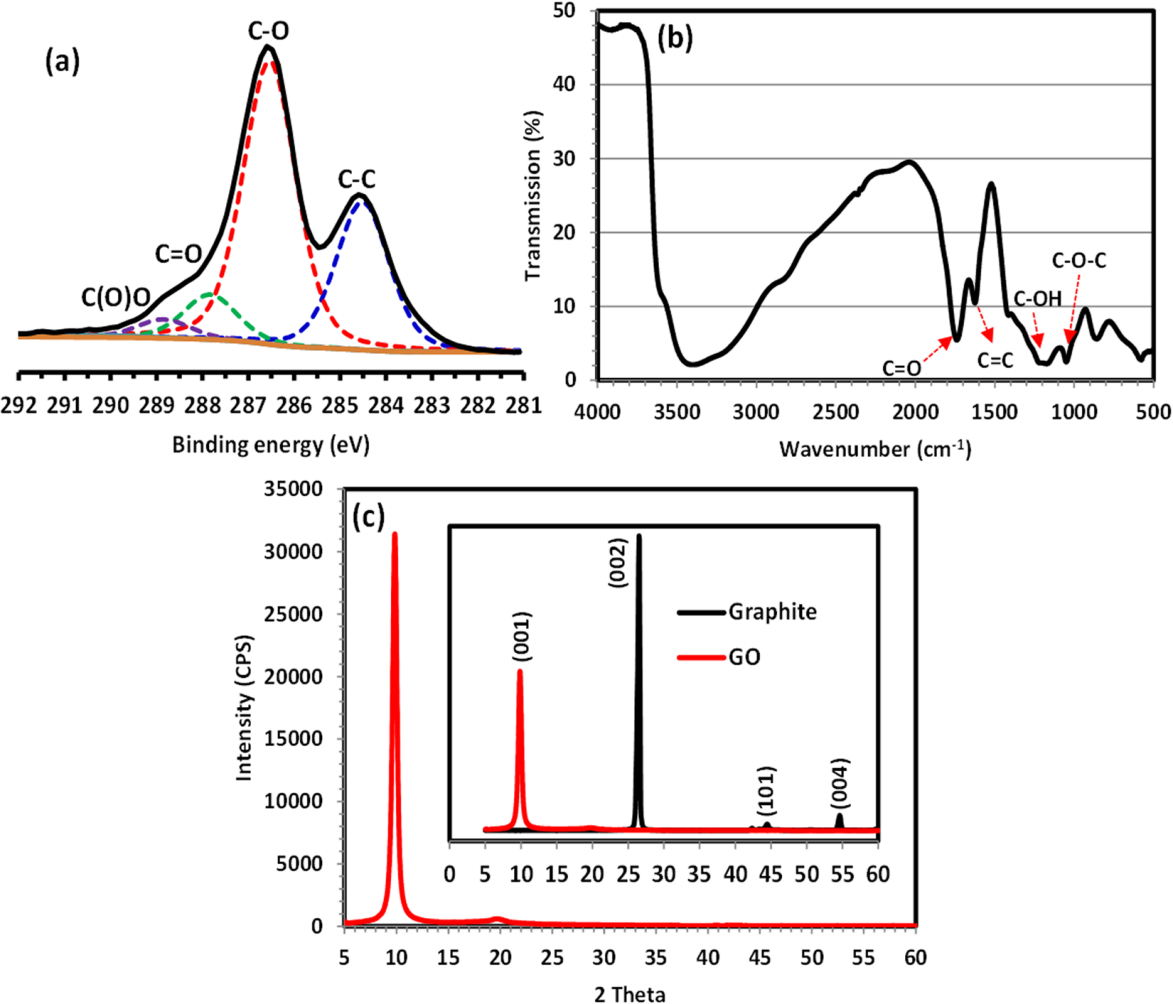
Surface properties of GO/PES membranes: (**a**) XPS deconvolution results of the C1s spectrum, (**b**) FT-IR spectrum, and (**c**) XRD patterns (inset indicates XRD patterns of graphite and GO film).

The elemental analysis of GO dispersion was performed by XPS and the O/C ratio of dry GO film found to be 0.48, which is somewhat higher than the values reported^6^. The high O content denotes a large number of oxygen-containing groups existing on the graphene sheet. The C1s spectrum and its deconvolution results are plotted in Fig. 2a. The relative peak area of the C-O groups was ~51% of the total C1s peak while the proportions of the C=O and C(O)O were 8% and 3%, respectively. The FT-IR spectrum of the GO/PES membrane indicated the presence of O-H groups (O-H stretching at 3400 cm^−1^ and C-O stretching at 1203 cm^−1^), epoxy groups (C-O-C stretching at 1048 cm^−1^), and carboxyl groups (C=O stretching at 1737 cm^−1^) as shown in Fig. 2b. In the XRD pattern (Fig. 2c) the intense (001) peak of the GO membrane material was located at 2θ = 9.86° (*d*-spacing: 8.97 Å), which agrees well with that reported in the literature^17^ and confirms a well-organized packing of GO sheets in the membrane. None of the characteristic peaks of the graphite starting material (e.g. the 100% peak at 2θ = 26.96° (002) [JCPDS file #04-014-0347] could be detected (Fig. 2c inset), which indicates that pure and highly oxidized GO was synthesized. The Raman spectrum of the GO surface, shown in Figure S1 in the Supplementary Information, reveals a high defect density. It shows that the GO/PES membrane synthesized was free of unexfoliated graphite.

**Figure 3:**
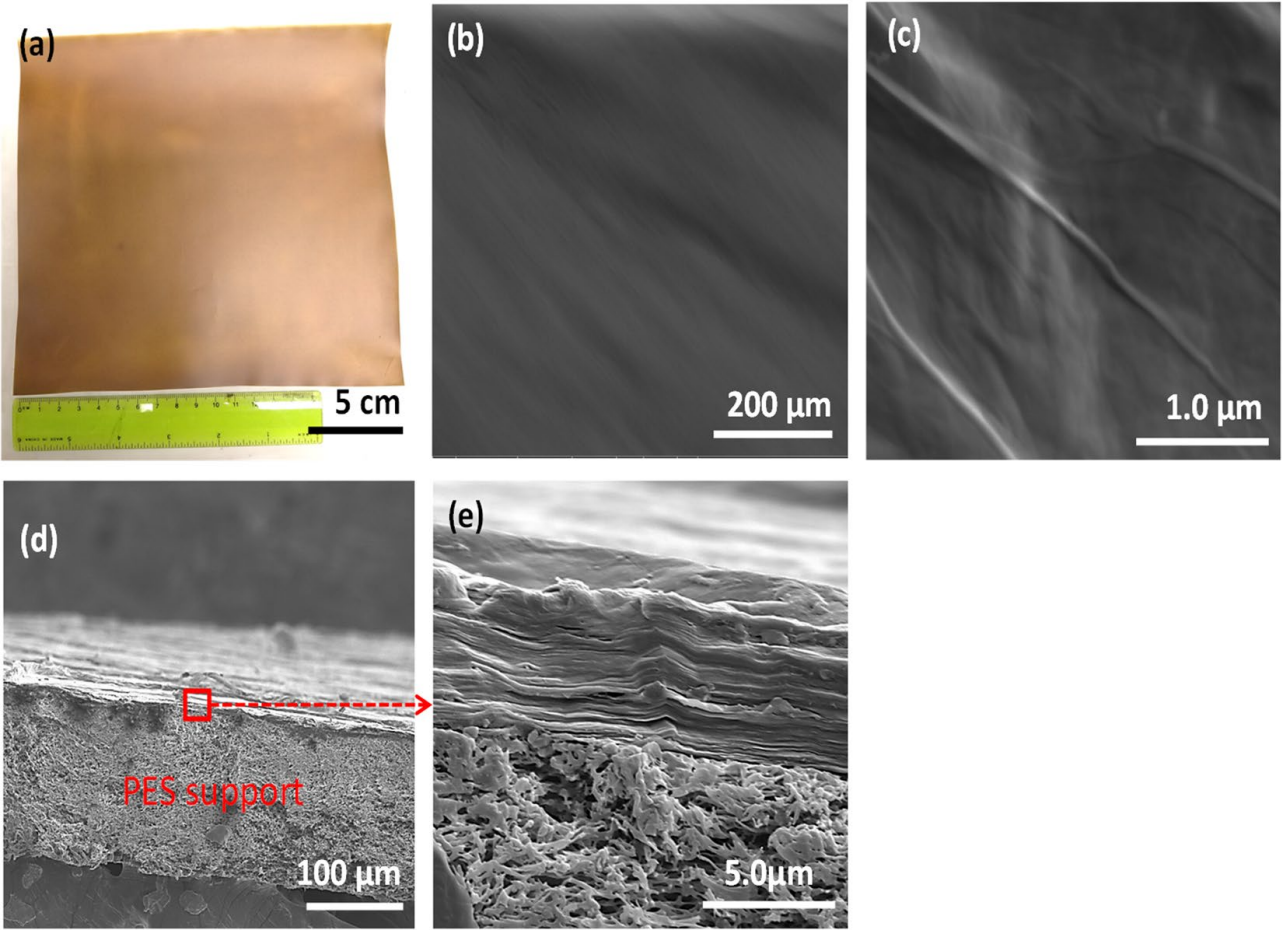
Morphology of the GO membrane observed by SEM: (**a**) digital photograph, (**b**) and (**c**) surface, and (**d**) and (**e**) cross-section.

It is evident from Fig. 3a that the flexible cast GO membrane on porous PES support was very uniform and homogeneous. The morphology of the GO membrane showed a very smooth surface, without obvious defects such as pores or cracks, as seen in Fig. 3b,c. The thickness was 5.0 ± 0.13 µm. The cross-section images, Fig. 3d,e, indicate well-ordered packed layers and the porous PES support that have a thickness of ~130 µm. The GO surface roughness determined by atomic force microscopy, shown in Figure S2 of the Supplementary Information, was 73.0 ± 21.3 nm. Roughness was calculated as root mean square of the measured film height over a defined surface area (10 × 10 µm). The surface morphology showed elongated, ridge-like domains. The roughness of particular ridges was very low (a few nanometers).

### Water-Ethanol Separation

**Figure 4:**
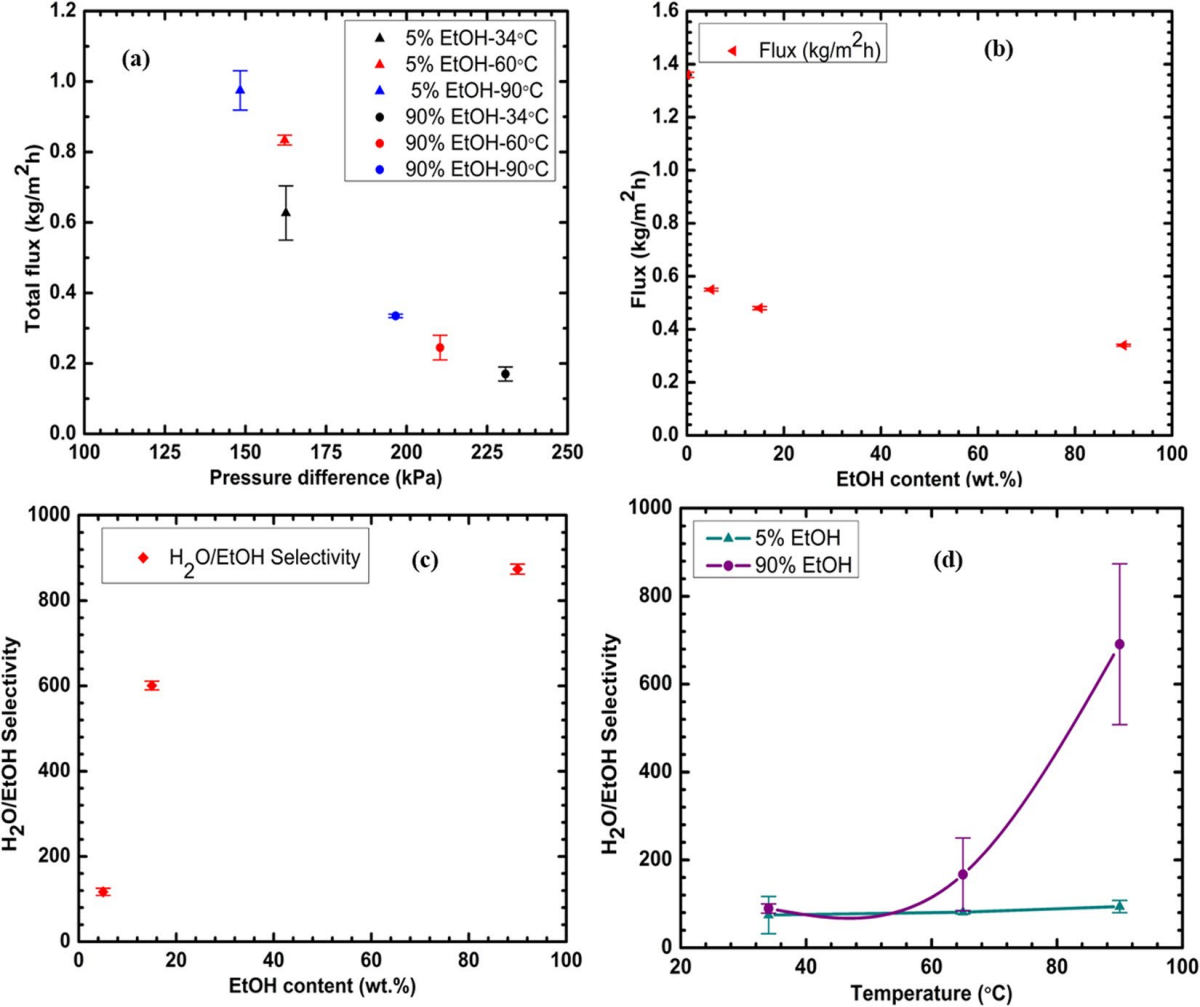
(**a**) Total flux as a function of pressure difference at different temperatures; (**b**) flux as a function of EtOH wt.%; (**c**) H_2_O/EtOH selectivity as a function of EtOH wt.%; and (**d**) the temperature dependence of H_2_O/EtOH separation selectivity of GO/PES membrane.

The performance of the GO/PES membrane for H_2_O-EtOH separation was tested by varying feed composition and separation temperature. To understand the effect of feed water content on the performance of the GO/PES membrane for H_2_O-EtOH separation, we varied the EtOH content in the H_2_O-EtOH feed mixture. In this study, we chose two distinct sets of feed composition: water-dominant (5% and 10% EtOH) and EtOH-dominant compositions (90% EtOH). Figure 4a show pressure differences between the two sides of the GO/PES membrane increases proportionally with the separation temperature (different symbol colors) and with the amount of EtOH in the feed (different symbol shapes). Interestingly, the total flux across the membrane exhibited a decrease as the pressure difference increased (Fig. 4a). This decrease might be due to several reasons including temperature polarization across the membrane^18^ and EtOH/water cluster formation. Our MD simulation results presented below shed light on the cluster formation. Figure 4b shows that the increase of EtOH content in the feed mixture at 90 °C from 0 wt.% to 90 wt.% led to a dramatic decrease in the total permeation flux from 1.36 kgm^−2^ h^−1^, when the feed consisted of 100% H_2_O (0 wt.% EtOH), to 0.3 kgm^−2^ h^−1^ when the feed was 90 wt.% EtOH. The permeation flux for 90 wt.% EtOH in the feed decreased from 0.33 kg m^−2^ h^−1^ at 90 °C to 0.15 kg m^−2^ h^−1^ at room temperature (not shown in this figure). In contrast, increasing the EtOH wt.% from 5 to 90 led to an increase in H_2_O/EtOH selectivity at 90 °C as illustrated by Fig. 4c. The H_2_O/EtOH selectivity increased from ~117 for 5 wt.% EtOH to a maximum selectivity of 874 when EtOH was 90 wt.% of the feed mixture. These results demonstrate that the present membrane is effective for separation of water and ethanol from mixtures with a wide range of water content.

The effect of temperature, in the range from 34 °C to 90 °C, on H_2_O/EtOH selectivity for EtOH wt.% of 5 and 90 is shown in Fig. 4d. There is a general trend of increase in the selectivity with increasing temperature. For 5 wt.% EtOH, the increase in selectivity is gradual from 74 at 34 °C to 94 at 90 °C. The selectivity increases much more dramatically from 90 at 34 °C to 690 at 90 °C for 90 wt.% EtOH. Lines are drawn merely to guide the eye. The results suggest that EtOH transport is severely impeded at high EtOH content and high temperature. According to the vapor-liquid equilibrium, mixtures of H_2_O and EtOH should be in the liquid phase at 65 °C and EtOH should be in the vapor phase at 90 °C. Separation at 65 °C and below could be regarded as pervaporation while that at 90 °C is vapor phase separation. EtOH vapor at 90 °C would mix poorly with liquid H_2_O molecules and would not effectively permeate the GO/PES membrane. This observation is consistent with previous reports in the literature of gas-tight GO membranes^5^. The present results show that the GO/PES membrane can be used for separation of H_2_O from a H_2_O-EtOH mixture over a wide range of operating temperatures and EtOH wt.%, in either pervaporation or vapor-phase mode.

The GO/PES membrane was stable up to 120 °C, and the H_2_O flux generally increased with an increase of separation temperature (see Figure S3 in the Supporting Information). The maximum H_2_O flux was 1.36 kg m^−2^ h^−1^ right after heating to 120 °C in 100% water feed. The flux returned to the initial level after the temperature was cooled down to 60 °C. These results demonstrate the response of the membrane to changes in the separation temperature and show the stability of the GO/PES membrane without GO delamination from the PES support, cracks, or leaks from the membrane under these separation conditions. Figure S3 in the Supporting Information represents a portion of the separation test performed at different temperatures and with feed changes for over a month (35 days) and repeated two more times. No cracks or holes were detected. Once the membranes were broken, the break could be detected by changes in the feed pressure and the flux. Cracks and holes were detected in free-standing GO membranes. In contrast, the GO/PES membranes were stable for over a month. The H_2_O flux decreased with an increase of EtOH content in feed and increased with an increase in the separation temperature. No decrease in the membrane selectivity was observed during the test period.

**Figure 5:**
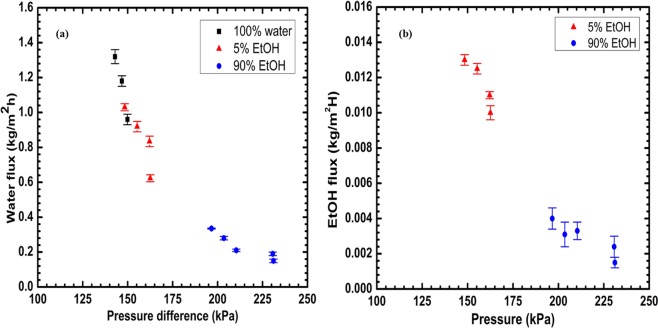
(**a**) Water and (**b**) EtOH fluxes as a function of pressure difference of GO/PES membrane. The Y-axis scale differs by a factor of 100 between these two plots.

Figure 5 represents water and EtOH fluxes as a function of the pressure difference between the two sides of GO/PES membrane. The water flux decreased with an increase of the proportion of EtOH in the feed. Both figures show that water and EtOH fluxes dramatically decreased with an increase in the pressure difference. One can invoke several mechanisms to explain these results. One of these mechanisms is impediment to the entry of molecules into the capillary by water-EtOH cluster formation. This effect may become more pronounced at higher EtOH content in the feed. This mechanism is supported by our simulation results presented below.

### Molecular simulation of water and ethanol transport

**Figure 6:**
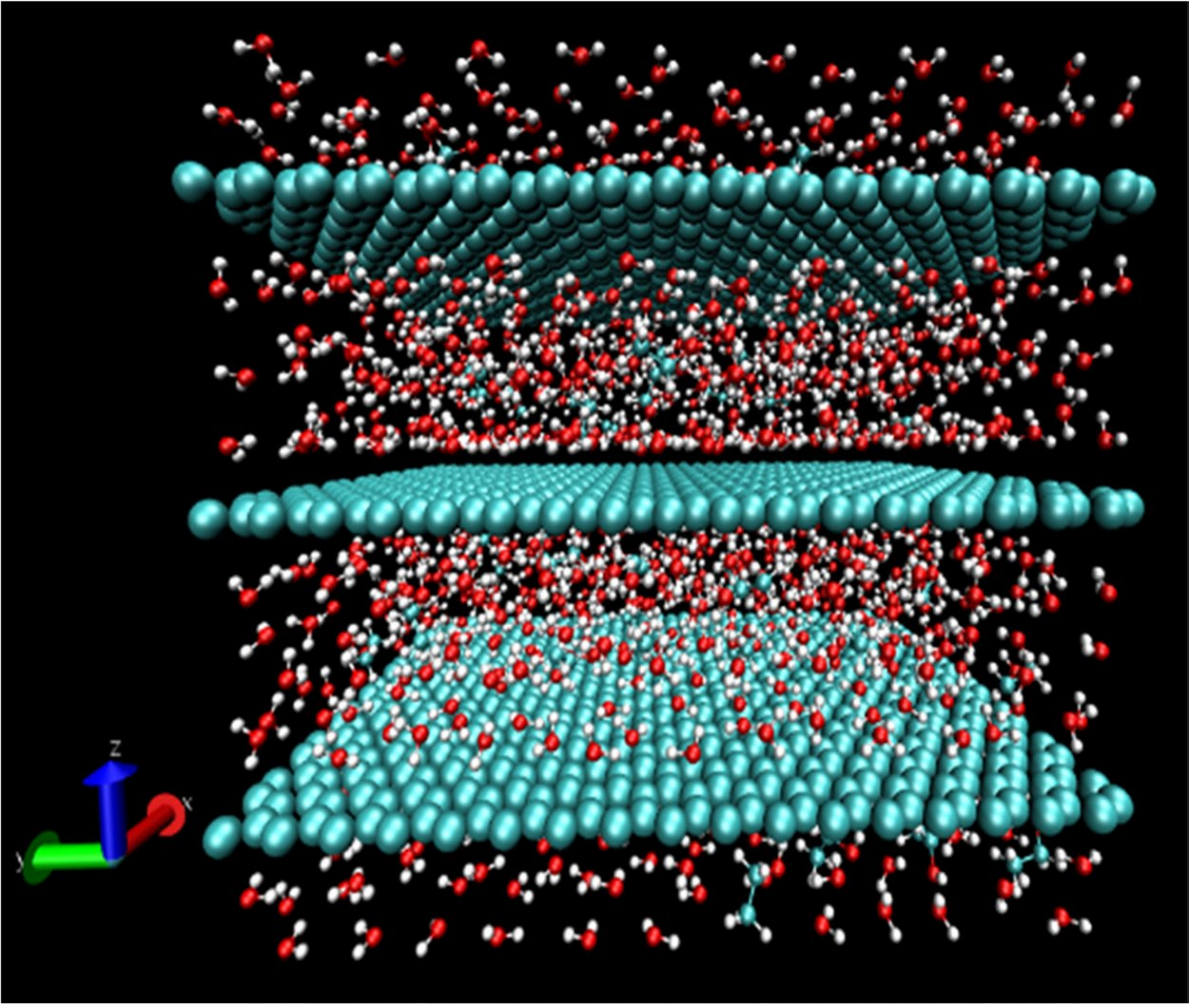
Snapshot of water-ethanol (90–10 wt.%) mixture in between graphene layers separated by 12 Å. O, H and C atoms are shown in red, white and cyan, respectively.

Figure 6 shows a representative snapshot of a 10 wt.% EtOH mixture between graphene layers separated by 12 Å at 300 K (27 °C). While we have previously simulated^19^ the transport of molecules intercalated between GO layers, we have considered only graphene capillaries here. It is well known that GO membranes have oxidized regions, graphitic regions and holes in various proportions depending on the method of preparation. It has been proposed by the Nair and coworkers^5,16^ that graphene capillaries play a key role in the preferential transport of water molecules. Our previous simulations also show that the transport of water through GO layers is too slow to account for the experimentally observed unimpeded permeation of water^5^.

The numbers of H_2_O and EtOH molecules in the system as a function of layer spacing and EtOH wt.% are listed in Table 1. The diffusion coefficients of water (*D*_*w*_) and ethanol (*D*_*e*_) as a function of layer spacing and EtOH wt.% are shown in Table 2. These spacings were chosen to cover a broad range of values from the case where water transport is known to be restricted (10 Å) from experiment^16^ to one where water shows bulk-like behavior (15 Å). Both *D*_*w*_ and *D*_*e*_ decreased with decreasing layer spacing. This finding is consistent with the recent experimental observation by Abraham *et al*.^16^, that water transport decreased for interlayer spacing below 10 Å. The negligible water transport seen in the present simulation for a spacing of 10 Å may be due to the presence of densely packed water molecules between the layers as seen in Fig. 6. It is also possible that molecular clusters could partially block the entry to the transport channels. For narrow spacing below 10 Å, water transport through carbon nanostructures has been shown^20^ to be in the form of a one-dimensional chain of molecules (single-file transport), which was not considered here.

**Table 1:** Number of water and ethanol molecules for layer spacings of 10, 12 and 15 Å.

Ethanol wt.%	Number of water molecules			Number of ethanol molecules		
	10 Å	12 Å	15 Å	10 Å	12 Å	15 Å
0	1110	1333	1665	0	0	0
10	1000	1199	1500	43	52	65
90	111	133	167	391	469	587

**Table 2:** Diffusion coefficients of H_2_O and EtOH for layer spacings of 10, 12 and 15 Å at 300 K.

Ethanol wt.%	H_2_O diffusion coefficient (10^−5^ cm^2^/s)			EtOH diffusion coefficient (10^−5^ cm^2^/s)		
	10 Å	12 Å	15 Å	10 Å	12 Å	15 Å
0	0.01^*^	1.55	1.80			
10	0.002^*^	0.30	1.29	0.003^*^	0.29	1.19
90	0.002^*^	0.11	0.53	0.003^*^	0.11	0.42

^*^Diffusion coefficients are too small to represent molecular transport over the time scale of the simulation.

For pure H_2_O (0 wt.% EtOH) between graphene layers separated by 12 or 15 Å, *D*_*w*_ was slightly less than the value of 2.3 × 10^−5^ cm^2^/s reported^21^ for bulk water. It was also an order of magnitude higher than values reported from simulation of water intercalated between GO layers^19^. These findings are consistent with the recent simulation results of Zheng *et al*.^22^ that the mobility of water molecules in the graphene channel was almost the same as that in bulk water, but the mobility of water molecules in GO channels was much lower. Water molecules can move almost unimpeded through graphene capillaries^5^ if the spacing is enough, but their movement is inhibited by hydrogen-bonded interactions in the space between GO layers^19^.

*D*_*w*_ in H_2_O-EtOH mixtures intercalated between graphene layers decreased with increasing EtOH content. The value of *D*_*w*_ in 10 wt.% EtOH mixture decreased from that in pure H_2_O (0 wt.% EtOH) by ~81% for a spacing of 12 Å and ~28% for a spacing of 15 Å. In 90 wt.% EtOH, the corresponding decrease was ~93% for a spacing of 12 Å and ~71% for 15 Å. Previous studies^23,24^ show that *D*_*w*_ in bulk H_2_O-EtOH mixtures decreases from that in bulk H_2_O by ~50% or less. The number of water molecules within the first coordination shell of EtOH, based on O-O distance of 3.3 Å, was 4.1, 3.3 and 2.95, for spacing of 10, 12 and 15 Å, respectively in the 10 wt.% EtOH mixture. The combined effects of spatial confinement and strong hydrogen-bond interaction in the binary mixture lead to the drastic decrease in *D*_*w*_. Zhang *et al*.^25^ have shown that clusters of water and alcohol could block molecular transport in binary mixtures.

The value of *D*_*e*_ decreased by about 65% in going from 10 wt.% EtOH to 90 wt.% EtOH for layer spacing of 12 Å and 15 Å. Such a large drop is inconsistent with the findings^23,24^ in bulk H_2_O-EtOH mixtures. The significant decrease in *D*_*w*_ and *D*_*e*_ with increasing EtOH wt.% can explain the experimentally observed decrease in flux with increasing EtOH content of the feed shown in Fig. 4. However, the differences between *D*_*w*_ and *D*_*e*_ for 10% EtOH and 90% EtOH at the layer spacing values studied were too small to account for the dramatic increase in H_2_O/EtOH selectivity observed experimentally. The simulations assume that both EtOH and H_2_O are present in the graphene capillary in the proportions listed in Table 1. We hypothesized that the H_2_O/EtOH selectivity is governed by preferential entry of water into the capillaries and not by relative differences in the diffusion of H_2_O and EtOH. With increasing EtOH content, H_2_O-EtOH clusters near the entrance of graphene capillaries, nanopores and slits between flakes are likely to hinder the entry of EtOH. The smaller H_2_O molecule may enter these spaces more readily.

**Figure 7:**
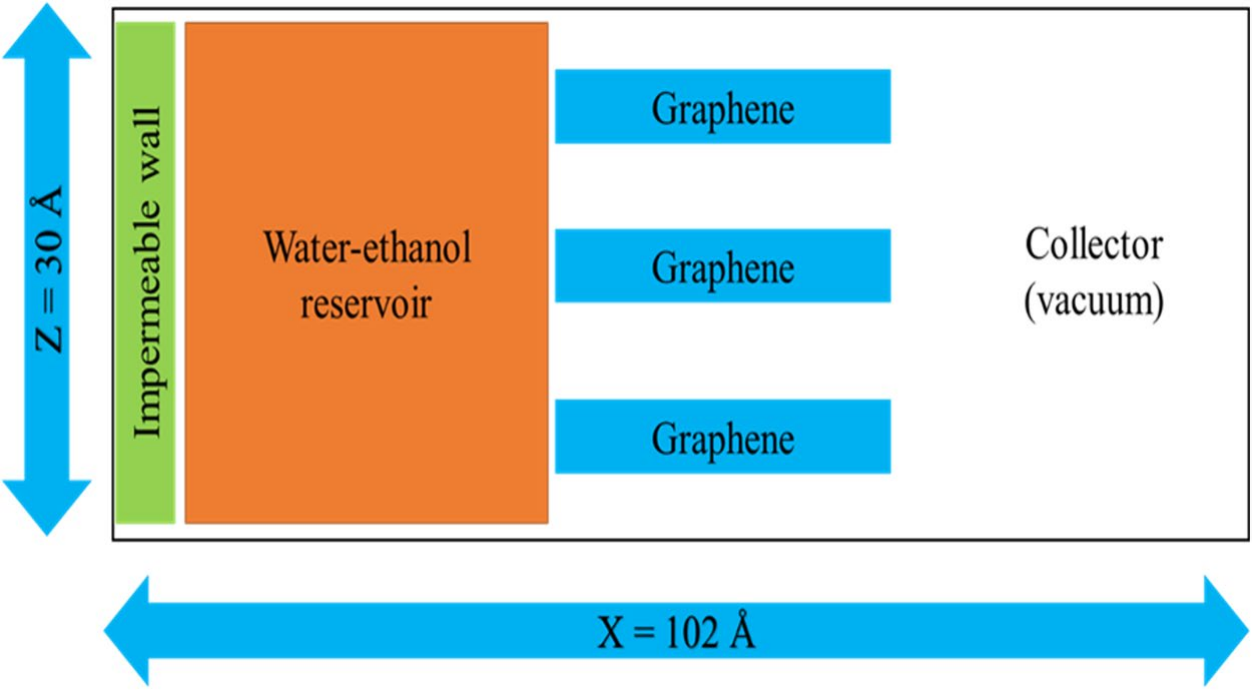
Schematic diagram of the simulated H_2_O-EtOH reservoir, graphene capillary and collector.

To test our hypothesis, we performed additional simulations of a model capillary system shown in Fig. 7. This system contained of a reservoir of H_2_O-EtOH mixture with a fixed graphene wall at one end to prevent molecules going across the boundary. This reservoir was separated from a collector initially at vacuum by graphene capillaries that had a fixed spacing. While we have simulated several spacings in the 10–15 Å range and mixtures with different proportions of EtOH, we will discuss here the findings for a spacing of 10 Å and EtOH content of 10 wt.% as an illustrative example.

**Figure 8:**
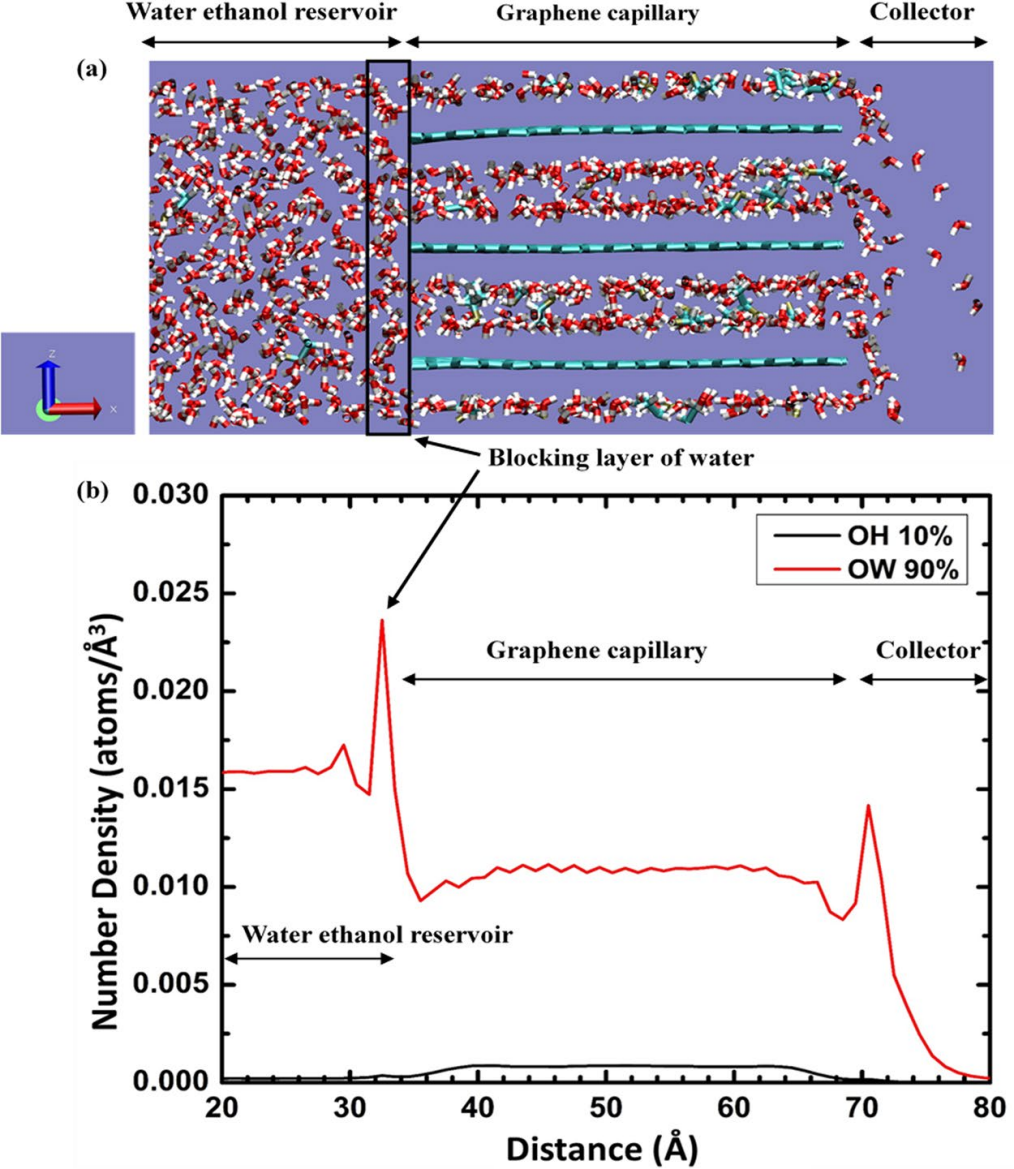
(**a**) Projection of a simulated configuration with 10 wt.% EtOH-90 wt.% H_2_O mixture in a 10 Å graphene capillary system. The dimensions of the simulation box in X, Y and Z direction are 102 Å, 42 Å and 30 Å, respectively. (**b**) The number density of oxygen atoms of ethanol (OH) and oxygen atoms of water (OW) along the X direction (capillary length) of the simulated box averaged over 20000 frames.

Figure 8a shows an orthographic projection of a simulated configuration corresponding to 10 wt.% ethanol and graphene capillaries with a spacing of 10 Å. A portion of the reservoir and collector are also shown. Water molecules (red and white) and ethanol molecules (tan, white and cyan) can be seen in the reservoir and capillaries. The molecules have entered the capillary and some water molecules can be seen exiting into the collector. At the entrance of the capillary on the left a layer, predominantly of water molecules in this case, forms in a way that impedes the entrance of molecules into the capillary. At higher EtOH concentrations (not shown here), this blocking layer has both EtOH and H_2_O molecules. Water molecules were observed to leave the capillary preferentially.

Further evidence of the blocking layer can be seen in the number density of molecules plotted in Fig. 8b. The number densities of oxygen of water (OW) and oxygen of ethanol (OH) are plotted along the X direction (length of the capillary) by averaging over 20000 configurations at 1 ps interval from a 20 ns trajectory. There is a sharp spike in the number density of water oxygen at the entrance of the capillary consistent with the molecular projection in Fig. 8a. Water molecules cluster at the entrance to the capillary. A smaller spike in the OW number density is also seen at the exit to the capillary. These clusters could impede large molecules and ions with large hydration shells from entering capillaries, pores and slits in GO membranes. This blocking effect may also happen to a certain extent at the capillary exit.

The findings of these simulations are consistent with the inference from the experiment of Nair *et al*.^5^ that water molecules may impede other molecules from moving through GO and the observation by Abraham *et al*.^16^ that the contribution of diffusion rates through graphene capillaries is smaller than the permeation effects in GO membranes. However, previous work^16,26^ has emphasized effects due to the size of the graphene channel. The present study shows that clusters at the entrance and exit of the channels can be influential in selective transport. Surface adsorption effects could play an important role in GO membranes but were not examined in this study. The simulated system is a highly simplified model of the experimentally studied membrane and the length and time scales of the simulation are short due to computational limitations. Nonetheless, the simulations provide an explanation for the experimentally observed selective transport of water and the decrease in flux with increasing proportion of ethanol in the feed. Future work could explore functionalization of pores in GO membranes to enhance the formation of clusters and control the selective transport of species.

The membrane synthesis presented in this study for water/EtOH separation offers ease of preparation and processing, stability in strong acids, alkali and organic solvents, stability of the pore size/interlayer spacing distribution, and the ability to tailor the interlayer spacing for further applications. The caveat is that much of the research on GO membranes has focused on fundamental science issues and lab scale studies. Scalability of the process, long-term membrane durability, resistance to fouling, and cost remain as practical challenges.

## Conclusions

We have used a simple casting method to prepare a stable PES-supported GO membrane (GO/PES) with excellent selectivity for the separation of water and ethanol. The GO/PES membrane showed several interesting features. First, it was very stable during the 120 hour-long testing period even at high operation temperature (120 °C). Second, it showed excellent H_2_O/EtOH selectivity over a wide range of EtOH wt.% in H_2_O-EtOH feed mixtures when the operation temperature was increased. Third, the GO/PES membrane can be used in either pervaporation mode or vapor-phase separation mode by controlling the operation temperature. Fourth, this membrane can be effective in the separation of water-contaminated bio-fuels. Molecular simulations revealed that the diffusion of H_2_O and EtOH between graphene layers decreases significantly with increasing EtOH content and thus provides an explanation for the decrease in flux with increasing EtOH wt.%. In addition, simulations revealed that clusters of predominantly water molecules form at the entrance and exit of graphene capillaries and impede the transport of ethanol. This blocking effect is a much greater contributor than differences in diffusion coefficients of the molecules to the observed selective transport of water over ethanol. These types of supported GO membranes that are stable at high temperature have applications in energy efficient separation of H_2_O in various chemical purification processes.

## Materials and Methods

### GO Synthesis and Membrane Preparation

Large graphite flakes (Asbury, cat #3763, 500 µm flakes) were oxidized using the method reported by Cruz-Silva *et al*.^27^ 5.0 g of graphite powder was added to the mixture of H_2_SO_4_ and H_3_PO_4_ (200/40 mL). To the reaction mixture, 24 g of KMnO_4_ was very slowly added under slow agitation with a Teflon rod. This exothermic reaction was maintained for 5 h with frequent agitation. The cooled thick paste was poured into 1.0 L of ice water with 5.0 mL H_2_O_2_ (30 wt.%), and kept overnight to consume the rest of the KMnO_4_ residue. The reaction mixture was centrifuged at 4000 rpm for 5 min, and the supernatant was decanted. The remaining solid material was poured into 1.0 L of 1.0 M H_2_SO_4_ solution to wash metal ions, the mixture was centrifuged at 4000 rpm for 10 min, and the supernatant decanted. Continuous washing with deionized water and centrifugation at 9,000 rpm for 30 min produced a two-layer structure. The bottom layer consisted of larger unexfoliated three-dimensional GO particles, while the top layer was a soft jelly-like dispersion that consisted mainly of exfoliated GO sheets. The top layer was collected and dispersed in a large amount of water (1 L). Large unexfoliated particles that might still be present were removed during another centrifugation step (4000 rpm for 3 min). The remaining GO was washed and separated by centrifugation; no solid pellet formed. Instead, the GO dispersion separated into a brown dispersion at the bottom of the vial and a clear supernatant that could be decanted. By centrifugation at 10,000 rpm for 40 min, the brown dispersion was concentrated into a slurry (pH > 5.0) containing about 1.0 wt.% of solid. The transparent GO dispersion was poured onto a PES membrane (0.2 micron, 200 × 200 mm, Lot#: 7046514, Sterlitech, Co. WA) and spread with a glass rod.

### Membrane Characterization

The X-ray diffraction (XRD) patterns of GO membranes were obtained on a Rigaku desktop X-ray diffractometer using Cu Kα (1.54059 Å) radiation with the X-ray generator operating at 20 kV and 30 mA. Data were collected for a 2θ range of 5.0–20.0° at an angular resolution of 0.01 °/s. The morphology of GO membranes was studied by scanning electron microscopy (SEM) using a JEOL microscope model JSM5900LV. Membrane samples for SEM analysis were loaded on carbon tape and plasma coated with Pt/Au for 240 s. A cross-sectional specimen was prepared by immersing the membrane in liquid N_2_ and snapping it in half. Fourier Transform Infrared (FT-IR) spectra were recorded over a range from 400–4000 cm^−1^ with a resolution of 4.0 cm^−1^, using a Nexus 670 FTIR spectrometer (Thermo Nicolet). X-ray photoelectron spectroscopy (XPS) measurements were performed using a Quantera Scanning X-ray Microprobe (Physical Electronics). This system used a focused monochromatic Al Kα X-ray (1486.7 eV) source and a spherical section analyzer. The instrument has a 32-channel detector. The X-ray beam used was a 100 W, 100 µm diameter beam that was rastered over a 1.2 mm by 0.1 mm rectangle on the sample. The X-ray beam was incident normal to the sample and the photoelectron detector was at 45° off-normal. High energy resolution spectra were collected using a pass-energy of 69.0 eV with a step size of 0.125 eV. For the Ag 3d5/2 line, these conditions produced a FWHM of 0.91 eV. The binding energy (BE) scale was calibrated using the Cu 2p3/2 line at 932.62 ± 0.05 eV and Au 4f7/2 at 83.96 ± 0.05 eV from known high purity references.

### Water-Ethanol Separation

The membrane performance was evaluated for H_2_O-EtOH separation under various conditions. Figure 1 shows a schematic diagram of the experimental setup. A GO/PES membrane (2 × 4 cm) backed by a metal form disk was packed into a planer testing cell made of stainless steel as shown in Fig. 1. The testing cell was placed inside a box oven (Yamato DV302) to maintain uniform temperature profile around the testing cell. Inside the rectangular testing cell, a metal mesh sheet, a corrugated plastic backing material sheet (with the flat against the sample), the membrane, and a silicon rubber “O-ring” were used to seal the feed and permeate chamber of the testing cell.

5–90 wt.% H_2_O in EtOH was pumped from a preheated feed tank into the testing cell. The feed stream was preheated by flowing it through a 20-foot long 1/8 in. stainless steel tube that was coiled and placed inside the oven. The coiled tube was used as a heat exchanger to ensure the feed mixture was heated to the testing temperature prior to entering the testing cell. The feed side temperature of the testing cell was monitored *in situ* with a thermocouple. After entering the testing cell, the feed stream passed over the membrane surface and flowed back into the tank. The liquid feed rate was at about 3 ml/min per square centimeter of membrane surface area and the feed pressure was slightly above atmospheric. The permeate side of the membrane cell was evacuated to ~10 Torr to provide a driving force for transport across the membrane. The permeate was collected in a liquid N_2_ cold trap and analyzed off-line with a high-performance liquid chromatograph (HPLC, Waters 2695) equipped with a refractive index detector (Waters 2414). The total permeation flux, permeability, and H_2_O-EtOH selectivity factor were calculated with the following equations based on experimental measurements:

1 $${J}_{m}=\frac{{W}_{p}}{(S{A}_{m})t}$$

2 $${P}_{i}=\frac{{F}_{i,p}}{(S{A}_{m})({\rm{\Delta }}pi)}$$

3 $${S}_{ij}=\frac{{({y}_{i}/{y}_{j})}_{p}}{{({x}_{i}/{y}_{j})}_{f}}$$

with *J*_*m*_ = permeation flux [kg/m^2^/h], *P*_*i*_ = permeance of species *i* [mol/m^2^/s/Pa], *S*_*ij*_ = separation factor of species *i* to *j*, *W*_*p*_ = amount of liquid condensed in the liquid N_2_ trap, *SA*_*m*_ = working surface area of the membrane [m^2^], *t* = testing duration time to collect *W*_*p*_ [s], *F*_*i*_ = permeation flow rate of species *i* [mol/s], *Δp*_*i*_ = partial pressure differential of species *i* between the feed and permeate sides [Pa], *y*_*i*_ = molar fraction of species *i* on the permeate side, *y*_*j*_ = molar fraction of species *j* on the permeate side, *x*_*i*_ = molar fraction of species *i* on the feed side, and *x*_*j*_ = molar fraction of species *j* on the feed side.

### Molecular Dynamics Simulations

Classical molecular dynamics (MD) simulations were performed to study H_2_O and EtOH intercalated between graphene layers of size 34.2 × 41.5 Å. The simulation cell contained three layers of graphene sheet. H_2_O-EtOH mixtures containing 0, 10, and 90 wt.% of ethanol were simulated. Three different layer separations were chosen between graphene layers, namely 10, 12 and 15 Å. The LAMMPS^28^ code was used to perform the MD simulations. An all-atom Optimized Potentials for Liquid Systems (OPLS) force field was employed to describe the bonded as well as non-bonded interactions for graphene and EtOH. The OPLS parameters for graphene and EtOH were adopted from the work of Tang *et al*.^29^ and Kahn *et al*.^30^, respectively. The F3C^31^ potential was used for water. The non-bonded interaction parameters of Wu and Aluru^32^ were used for the graphene-H_2_O interactions. The geometric combination rule was used to determine the non-bonded interaction parameters for graphene-EtOH and H_2_O-EtOH interactions.

The model simulated a graphene capillary and did not include functional groups. Harmonic restraints were applied to the initial position of every carbon atom in the graphene layer using a force constant to maintain a constant capillary diameter. The production run was performed at constant volume and temperature (NVT ensemble) at a temperature of 300 K for 2 ns with a time step of 1 fs. Coordination numbers and diffusion coefficients were determined over 2000 frames of the trajectory at an interval of 1 ps. A H_2_O molecule was considered a member of the first hydration shell of EtOH if the oxygen atom of H_2_O was within 3.3 Å from the oxygen atom of EtOH molecule^33^. The diffusion coefficients of H_2_O and EtOH were calculated from a linear regression to the variation of mean square displacement with time.

To study the preferential entry of water molecules in the graphene capillary, we simulated a different system containing a H_2_O and EtOH reservoir, graphene layers with a 10 Å spacing and a vacuum region that served as a collector for transported molecules. The reservoir had 10 wt. % EtOH. The reservoir had 1193 molecules of water and 52 molecules of ethanol. The dimension of the graphene layers in X and Y direction were 33.65 Å and 40.54 Å, respectively. Each graphene layer forming the capillary had 544 C atoms. The carbon atoms of the capillary in the X direction were terminated by H atoms. We used an impermeable graphene wall to hold the solution in the reservoir and prevent direct exchange between the reservoir and collector. The dimensions of the simulated system in X, Y and Z direction are 102 Å, 42 Å and 30 Å, respectively. Periodic boundary condition was maintained in all directions. A schematic diagram of the simulated system is presented in Fig. 7. We used the same force field as in the previous simulation. The impermeable membrane, graphene capillary and a few water molecules in front of the capillary channel were position restrained for NVT equilibration run up to 1 ns with a time step of 1 fs at 300 K temperature. For the production run, only the impermeable membrane and graphene capillary were position restrained. The solution could move through the capillary and onward to the collector region. The production run was performed with the NVT ensemble at a temperature of 300 K for 20 ns with a time step of 1 fs. The number densities of H_2_O and EtOH molecules were calculated along the X direction of the simulation box by taking the average over 20,000 frames from a 20 ns trajectory at a time interval of 1 ps. For the number density calculation, the bin width was 1 Å along the X direction.

## Supplementary information

Supplementary Information
