# Supplementary material for: Highly Selective Supported Graphene Oxide Membranes for Water-Ethanol Separation
Source: Sci Rep. 2019 Feb 19;9:2251. doi: 10.1038/s41598-019-38485-y (PMC6381104; doi:10.1038/s41598-019-38485-y)
Supplement: Supplementary file 1 — Supplementary Information [file 41598_2019_38485_MOESM1_ESM.docx]

Highly Selective Supported Graphene Oxide Membranes for Water-Ethanol Separation

Yongsoon Shin, Mohammad Fuad Nur Taufique, Ram Devanathan, Erika C. Cutsforth, Jaewon Lee, Wei Liu, Leonard S. Fifield, and David W. Gotthold*

902 Battelle Blvd, P.O.Box 999, Pacific Northwest National Laboratory, Richland, Washington 99352, United States.

*Corresponding author: David.gotthold@pnnl.gov

**Figure S1**. Raman spectrum of the GO/PES membrane used in this study.

**Figure S2.** Atomic force microscopy image (top) and roughness profile (bottom) of GO surface in the GO/PES membrane: solid red and blue lines indicate roughness profiles at different locations. Roughness calculated as root mean square of the measured film height over a defined surface area (10 µm x 10 µm) was 73.0 ± 21.3 nm.

**Figure S3.** Illustration of the stability of the GO/PES membrane during continuous testing under a variety of conditions.
